# Machine learning for characterizing risk of type 2 diabetes mellitus in a rural Chinese population: the Henan Rural Cohort Study

**DOI:** 10.1038/s41598-020-61123-x

**Published:** 2020-03-10

**Authors:** Liying Zhang, Yikang Wang, Miaomiao Niu, Chongjian Wang, Zhenfei Wang

**Affiliations:** 1 School of Information Engineering, Zhengzhou University, Zhengzhou, Henan P.R. China; 2 Department of Epidemiology and Biostatistics, College of Public Health, Zhengzhou University, Zhengzhou, Henan P.R. China

**Keywords:** Epidemiology, Risk factors

## Abstract

With the development of data mining, machine learning offers opportunities to improve discrimination by analyzing complex interactions among massive variables. To test the ability of machine learning algorithms for predicting risk of type 2 diabetes mellitus (T2DM) in a rural Chinese population, we focus on a total of 36,652 eligible participants from the Henan Rural Cohort Study. Risk assessment models for T2DM were developed using six machine learning algorithms, including logistic regression (LR), classification and regression tree (CART), artificial neural networks (ANN), support vector machine (SVM), random forest (RF) and gradient boosting machine (GBM). The model performance was measured in an area under the receiver operating characteristic curve, sensitivity, specificity, positive predictive value, negative predictive value and area under precision recall curve. The importance of variables was identified based on each classifier and the shapley additive explanations approach. Using all available variables, all models for predicting risk of T2DM demonstrated strong predictive performance, with AUCs ranging between 0.811 and 0.872 using laboratory data and from 0.767 to 0.817 without laboratory data. Among them, the GBM model performed best (AUC: 0.872 with laboratory data and 0.817 without laboratory data). Performance of models plateaued when introduced 30 variables to each model except CART model. Among the top-10 variables across all methods were sweet flavor, urine glucose, age, heart rate, creatinine, waist circumference, uric acid, pulse pressure, insulin, and hypertension. New important risk factors (urinary indicators, sweet flavor) were not found in previous risk prediction methods, but determined by machine learning in our study. Through the results, machine learning methods showed competence in predicting risk of T2DM, leading to greater insights on disease risk factors with no priori assumption of causality.

## Introduction

Type 2 diabetes mellitus (T2DM) is a long-term metabolic disorder with high morbidity in humans around the world. The prevalence of diabetes is increasing rapidly worldwide, including in China^1^. In China, diabetes was estimated to affect 144.4 million people aged 20–79 according to the report of the international diabetes federation in 2017^2^. The prevalence of diabetes in a rural population of Henan province is high which can be seen in the Henan Rural Cohort Study^3^. Although diabetes is an irreversible disease, it is largely preventable^4^. The risk of developing diabetes will be reduced through early detection and lifestyle interventions. For individual patient care, physicians are well prepared to identify those at risk for T2DM. However, when trying to screen thousands of patients with high-risk conditions, the challenges faced by physicians become apparent. There is a need for analytics techniques to assist in T2DM mass screening.

Many risk scores based on statistical knowledge have been developed for predicting individual’s risk of developing T2DM, such as risk evaluation formula^5^, Archimedes trial-validated diabetes model^6^, the diabetes risk score^7^, genetic risk score^8^, the New Chinese Diabetes Risk Score^42^ and the American Academy of Family Physicians risk model^9^. These methods made the implicit assumption that each risk factor was linear to the outcome. The complex relationships between nonlinear interaction factors might be oversimplified, leading to the potential loss of related information^10,11^. Moreover, when the number of variables increased, the hypothesis testing method became complicated^12^. In contrast to traditional methods, machine learning can learn the nonlinear interactions iteratively from large amounts of data using computer algorithms^13^, which have been applied in various fields, such as disease risk assessment and prediction^14,15^. Recent research shows that machine learning methods can describe patients’ characteristics and identify patients at risk of developing T2DM^16,17^. A study illustrated the performance of support vector machine for detecting persons with diabetes and pre-diabetes^18^. To assess the ability to estimate the risk of developing T2DM, a study evaluated the performance of different machine learning and statistical techniques, and the experimental results showed the comprehensive performance the ensembles of ANN was better than other models^19^. A data mining pipeline based on classification algorithm was built to predict T2DM complications based on electronic health record data from nearly one thousand patients, which showed the validity of machine learning method^20^. An ensemble approach with the use of the vote method with three Decision Trees was developed to predict incident diabetes using 13 attributes^21^, and improved the value of AUC to 0.922. A novel joint clustering and classification (JCC) method which could discover hidden clusters features in the patient samples was developed to predict diabetes, and the method performed best among the methods that were applicable to the interpretation of prediction^22^. A study used neural network, decision tree, and random forest to predict diabetes mellitus with 14 attributes, and the results showed that the highest accuracy method was random forest^23^. Another study compared the performance of several machine learning techniques to predict the risk of developing T2DM in short, medium, and long term, and the results showed that logistic regression outperformed in short, medium term while support vector machines presented better performance in long term^24^. A machine learning-based framework for identifying subjects with T2DM from EHR was constructed via feature engineering, and the results revealed that the framework performed higher identification compared with the expert algorithm^25^.

However, the current methods just focused on performance comparison of prediction techniques with fixed number of variables, and they were also done on a small population sample. To date, there has been no large-scale investigation applying machine-learning for risk assessment in the general rural population. Therefore, the purpose of this study was to (1) evaluate an array of machine learning algorithms for predicting the risk of T2DM in a rural Chinese population; (2) identify the important variables, and (3) reveal the model performance of each model on a varying number of variables.

## Method

### Study participants

The participants of this study came from the Henan Rural Cohort Study (Registration number: ChiCTR-OOC-15006699). A total of 39259 participants aged between 18 to 79 years were recruited from five rural areas in Henan province of China over the period between July 2015 and September 2017. The design and population characteristics of the study have been described in the previous articles^26–28^. Data on socio-demographic characteristics, information on physical examination, and laboratory test data were collected. Participants were excluded if they: (1) were diagnosed with kidney failure (N = 18) or cancer (N = 332); (2) had type 1 diabetes mellitus (N = 4); (3) had gestational diabetes mellitus (N = 634); (4) had incomplete information on diagnoses of T2DM (N = 63); and (5) had incomplete information of potential covariates (n = 2127). Finally, 36,652 participants were included for the present study.

### Definition of T2DM

After excluding participants with type 1 diabetes mellitus, gestational diabetes mellitus, and other special type diabetes, T2DM was a self-reported previous diagnosis of diabetes by a physician or fasting plasma glucose level ≥7.0 mmol/L according to the American Diabetes Association (ADA) diagnostic criteria^29^.

### Machine learning methods

We used logistic regression, artificial neural networks, classification and regression tree, support vector machine, and ensemble learning (random forest and gradient boosting machine) to build the risk assessment model. From the description of basic characteristics of non-T2DMs and T2DMs, the data is imbalanced. The model is likely to be biased towards the dominant class, with poor accuracy in classifying negative cases. In view of this problem, the Synthetic Minority Over-Sampling Technique (SMOTE)^24,30,31^ algorithm was used to address the data. All models were constructed using the package sklearn (0.21.3) of Python 3.7 programing language.

### Artificial neural networks

Artificial neural networks^32^ are computing systems that are based on the neurons of the human brain. ANN can learn all complex and non-linear interactions between variables to look for patterns in the data. ANN is divided into multi-hidden layer neural network and single hidden layer neural network. Each layer contains a number of neurons connected by directed arcs with variable weights. In our study, the neural network consists of three layers: an input layer to accept all risk factors, a hidden layer to process information and an output layer to calculate responses.

### Classification and regression tree

A decision tree is a tree structure in which each internal node represents a test on an attribute, each branch represents a test output, and each leaf node represents a category^33^. Typical algorithms of decision tree include ID3, C4.5, CART, and so on. Considering the extensive application of CART in clinical and basic research, we used CART in this study^34^. CART is a non-parametric decision tree learning technology, which generates a classification tree or regression tree according to whether the dependent variable is classified or numerical^35^.

### Logistic regression

Logistic regression (LR) is a generalized linear regression analysis model, which works to find the best fitting model that can describe the relationship between dependent variables and independent predictors^36^. LR model is most widely used when people are interested in predicting disease or health status^37^. The LR model can compute the probability of an individual developing T2DM based on the risk factors input. If a subject suffers from T2DM, the value of Y is 1; otherwise, Y is 0. We defined the probability of an individual developing T2DM is $\,p(Y=1|X)=p(X)$. Then, the formula of the LR model is defined as follows.

1 $$logit(p)=ln\,\left[\frac{p(X)}{1-p(X)}\right]={\beta }_{0}+{\beta }_{1}{X}_{1}+{\beta }_{2}{X}_{2}+\cdots +{\beta }_{k}{X}_{k}$$

and equivalently, after exponentiating both sides:

2 $$\frac{p(X)}{1-p(X)}={e}^{{\beta }_{0}+{\beta }_{1}{X}_{1}+{\beta }_{2}{X}_{2}+\cdots +{\beta }_{k}{X}_{k}}$$

The probability of an individual developing T2DM is

3 $$p(X)=\frac{{e}^{{\beta }_{0}+{\beta }_{1}{X}_{1}+{\beta }_{2}{X}_{2}+\cdots +{\beta }_{k}{X}_{k}}}{1+{e}^{{\beta }_{0}+{\beta }_{1}{X}_{1}+{\beta }_{2}{X}_{2}+\cdots +{\beta }_{k}{X}_{k}}}$$

Where $X=({X}_{1},{X}_{2}\cdots {X}_{k})$ represents the risk factors, $\beta =({\beta }_{1},{\beta }_{2}\cdots {\beta }_{k})$ are the coefficients estimated by using the method of maximum likelihood.

### Support vector machine

Support vector machine (SVM) is a kind of generalized linear classifier that classifies binary data according to supervised learning. Its decision boundary is the maximum margin hyper plane for the positive and negative classes^38^. In our study, each data sample is made of 60 features. The value of each feature is a vector of a particular dimension. Then, we used SVM to construct a hyperplane in a high-dimensional space, which can distinguish the two classes nicely.

### Ensemble learning

Ensemble learning is an algorithm that combines basic learners such as decision trees and linear classifiers. The main idea of ensemble learning is to use multiple learning algorithms to achieve better performance than any constituent learning algorithm alone. Common types of ensembles are boosting, bagging, random subspace.

Random forest (RF) is an algorithm combines bagging ensemble learning theory with random subspace approach. RF generates many decision trees for splitting data randomly at training time. For each node of the base decision tree, a subset containing K attributes is randomly selected from the attribute set of that node, and then an optimal attribute is selected from the sub-set for partitioning. Each tree provides a classification as a vote for each tree, and the RF ultimately chooses the classification with the most votes^39^.

Gradient boosting machine (GBM) is an iterative algorithm whose core idea is to train different classifiers (weak classifiers) for the same training set, and then combine these weak classifiers to form a stronger final classifier (strong classifier). Through a series of iterations to optimize the classification results, each iteration is introduced into a weak classifier, to overcome the existing shortcomings of weak classifier combination. GBM is based on the residual of training data fitted by the previous weak classifier to enhance the model when training each weak classifier. Compared with most learning algorithms, it is less prone to over fitting.

**Figure 1:**
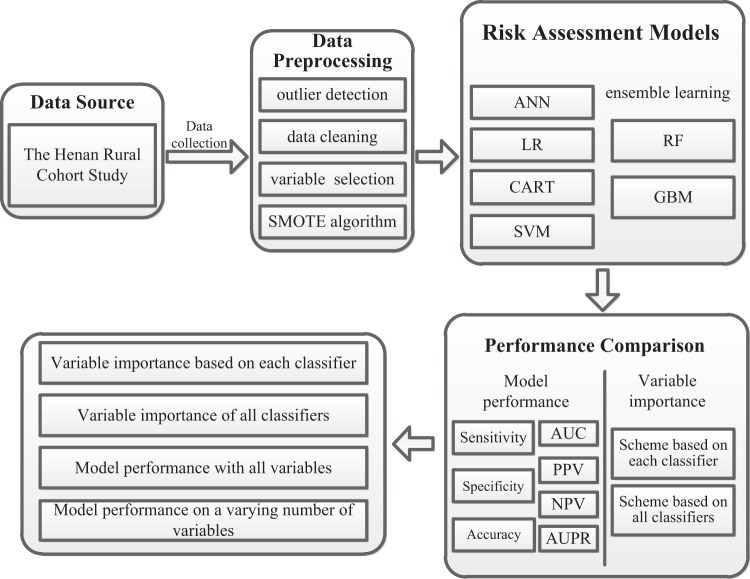
Methodology. Abbreviation: LR, logistic regression; CART, classification and regression tree; GBM, gradient boosting machine; ANN, artificial neural network; RF, Random forest; SVM, Support vector machine; PPV, positive predictive value; NPV, negative predictive value; AUC, area under curve; AUPR, area under precision recall curve.

Figure 1 showed the methodology of this study. In this study, risk assessment models for T2DM were developed using 6 ML algorithms on all variables. Next, algorithms were iteratively introduced to a growing number of ranked variables (5/10/15/…) selected by the algorithm itself. All models were trained and tested by 10-fold cross-validation during each iteration process, which was repeated 100 times. Performance of all models was calculated on the test samples. All models’ parameters were determined using 10-fold cross-validation and grid search on the training data (Supplementary Table 2).

### Statistical analysis and evaluation on the model

Model performance: Discrimination refers to the model’s ability to identify who is at risk of developing T2D and who is not. We used sensitivity, specificity, positive predictive value (PPV), negative predictive value (NPV), area under precision recall curve (AUPR) and area under curve (AUC) to evaluate discrimination. Sensitivity is a synonym for recall rate, true positive rate, and represents the proportion of real positive samples that are identified correctly. For instance, in our study, the subject who diagnosed with T2DM was defined as 1, namely the positive sample. Otherwise, it was a negative sample (0). Specificity indicates the rate of real negative samples can be detected correctly. PPV stands for the proportion of positive results in diagnostic tests that are true positive results. NPV is the proportion of negatives in diagnostic tests that are true negative results. For binary classification models, AUC and AUPR were also used to evaluate the performance.

Variable importance: For the diabetes study, we also listed the importance of variables. For LR and SVM models, variable importance was determined by the coefficient effect size. CART model estimated the variable importance by summing changes in the mean squared error due to splits on every variable and dividing the sum by the number of branch nodes. Variable importance of RF was estimated by permutation of out-of-bag variable observations. GBM computed the variable importance by summing these estimates over all weak learners in the classification ensemble method. ANN used overall connection weights of variable to filter variables^40^. In order to combine the variable importance of each method, the variable importance was also estimated using shapley additive explanations approach, which is a unified approach to explain the output of any machine learning model^41^.

Categorical variables were described as percentages (%), and continuous variables were shown as mean ± standard deviation (SD). Differences in the characteristics of T2DM and Non-T2DM groups were determined with the chi-square test for categorical variables and t-test for continuous variables. All statistical analyses were performed using SPSS (v.21, IBM) and a two-tailed P value < 0.05 was considered statistically significant.

### Ethics approval

Ethics approval was obtained from the “Zhengzhou University Life Science Ethics Committee”, and written informed consent was obtained for all participants. Ethic approval code: [2015] MEC (S128). The present study was conducted in accordance with the guidelines of the Declaration of Helsinki.

## Results

### Basic characteristics

The general characteristics of the study population were presented in Table 1. The study population consisted of 14,375 men and 22,277 women. Compared with participants without T2DM, individuals with T2DM tended to be with higher age, BMI, waist circumference, heart rate, waist to height ratio, urine glucose, low-density lipoprotein cholesterol, and were more likely to have a family history of T2DM, hypertension, coronary heart disease. In contrast, among participants without T2DM, higher creatinine, higher high-density lipoprotein cholesterol, and higher uric acid were more common. Further details were presented in Supplementary Table 1.

**Table 1:** General characteristics of the study population.

Variable	Total (n = 36652)	Non-T2DM (n_1_ = 33296)	T2DM (n_2_ = 3356)	*P-Value*
Age (years)	55.60 ± 12.17	55.11 ± 12.32	60.51 ± 9.20	<0.001
Men, n (%)	14375(39.22)	13114(39.39)	1261(37.54)	0.040
Education, n (%)				<0.001
≤Primary school	16432(44.83)	14567(43.75)	1865(55.57)	
Middle school	14614(39.87)	13507(40.57)	1107(32.99)	
≥High school	5606(15.30)	5222(15.68)	384(11.44)	
Marry, n (%)				0.027
Married/cohabitating	32927(89.84)	29949(89.95)	29877(88.74)	
Divorced/widowed/unmarried	3725(10.16)	3347(10.05)	378(11.26)	
Average monthly individual income, n (%)				<0.001
<1000	25111(68.51)	22709(68.20)	2402(71.57)	
1000~	8833(24.10)	8083(24.28)	750(22.35)	
≥2000	2708(7.39)	2504(7.52)	204(6.08)	
High fat diet, (≥75 g/day)	7088(19.34)	6544(19.65)	544(16.21)	<0.001
Sweet flavor, n (%)				<0.001
No	15872(43.30)	13495(40.53)	2377(70.83)	
Mild	14217(38.79)	13500(40.55)	717(21.36)	
Middle	5720(15.61)	5494(16.50)	226(6.73)	
Heavy	843(2.30)	807(2.42)	36(1.07)	
Waist circumference (cm)	84.13 ± 10.33	83.62 ± 10.22	89.32 ± 10.01	<0.001
Body mass index (kg/m^2^)	24.85 ± 3.53	24.72 ± 3.49	26.20 ± 3.62	<0.001
Waist to hip ratio	0.89 ± 0.07	0.88 ± 0.07	0.93 ± 0.07	<0.001
Pulse pressure (mm Hg)	48.25 ± 13.08	47.72 ± 12.85	53.45 ± 14.22	<0.001
Heart rate (beats/min)	75.72 ± 11.12	75.34 ± 10.94	79.54 ± 12.13	<0.001
Total cholesterol (mmol/l)	4.75 ± 0.97	4.72 ± 0.95	5.01 ± 1.11	<0.001
Triglyceride (mmol/l)	1.68 ± 1.12	1.64 ± 1.07	2.13 ± 1.44	<0.001
HDL-C (mmol/l)	1.32 ± 0.33	1.33 ± 0.33	1.23 ± 0.32	<0.001
LDL-C (mmol/l)	2.87 ± 0.81	2.85 ± 0.80	3.06 ± 0.93	<0.001
Insulin (ug/l)	10.85 ± 5.30	10.69 ± 5.04	12.51 ± 7.19	<0.001
Creatinine (umol/L)	62.07 ± 14.00	62.31 ± 13.75	59.61 ± 16.08	<0.001
Uric acid(umol/L)	286.50 ± 79.29	287.77 ± 79.19	273.87 ± 79.22	<0.001
Urinary protein, n (%)	1087(2.97)	797(2.39)	290(8.64)	<0.001
Urine glucose, n (%)	915(2.50)	125(0.38)	790(23.54)	<0.001
Hypertension, n (%)	11943(32.58)	10225(30.71)	1718(51.19)	<0.001
Coronary heart disease, n (%)	1620(4.42)	1368(4.11)	252(7.51)	<0.001
T2DM history of mother, n (%)	1070(2.92)	813(2.44)	257(7.66)	<0.001
T2DM history of father, n (%)	532(1.45)	432(1.30)	100(1.45)	<0.001

Abbreviations: SD, standard deviation; HDL-C, high-density lipoprotein cholesterol; LDL-C, low-density lipoprotein cholesterol; T2DM, type 2 diabetes mellitus.

### Variable importance analysis

The top 10 variables according to the variable importance of each algorithm were presented in Table 2 (Supplementary Table 3). Elevated urine glucose level was presented as top-ranked variables by all algorithms. Indicators of obesity repeatedly were appeared at the top of the list, such as waist to hip ratio, and waist to height ratio. This phenomenon confirmed that obesity is a risk factor of T2DM. Hypertension was ranked as an important factor of T2DM by most models, perhaps reflecting the relationship between hypertension and the development of T2DM. The risk factors in the New Chinese Diabetes Risk Score included sex, age, family history of diabetes, waist circumference, BMI, SBP. Several risk factors of the New Chinese Diabetes Risk Score (age, family history of diabetes, sex, and SBP) were shown in the list of top-ranked variables in our study. Common variables for diabetes were also identified by machine learning methods, such as genetic factors, hypertension, insulin, and so on. Also, new important variables (urinary parameters) were not found in previous risk prediction methods but determined by machine learning. Furthermore, the LR, SVM and ANN models prioritized genetic factor and urinary parameters, such as T2DM history of mother/father, urine glucose, urine protein, and so on.

**Table 2:** The top-10 ranked variables by the variable importance for each algorithm.

Rank	Machine-learning algorithms					
	LR	CART	GBM	ANN	RF	SVM
1	Urine glucose	Urine glucose	Urine glucose	Urine glucose	Urine glucose	Urine glucose
2	Diabetes history of mother	Sweet flavor	Sweet flavor	Diabetes history of mother	Sweet flavor	Urinary protein
3	Urinary protein	Sour flavor	Waist to hip ratio	Urinary protein	Waist to hip ratio	Diabetes history of mother
4	Diabetes history of father	Waist to hip ratio	Hypertension	Urine latent blood	Age	Diabetes history of father
5	Urine ketone bodies	Age	More vegetables and fruits	Sweet flavor	Creatinine	Urine ketone bodies
6	Hypertension	Diabetes history of mother	Age	Diabetes history of father	Uric acid	Hypertension
7	Coronary heart disease	Waist to height ratio	Urinary vitamin C	Urine ketone bodies	Heart rate	Coronary heart disease
8	Low-density lipoprotein cholesterol	Insulin	Urine PH	Gender	Insulin	Low-density lipoprotein cholesterol
9	Urine PH	Pulse pressure	Sour flavor	Systolic blood pressure	Triglyceride	Urine PH
10	Urine nitrite	Heart rate	Diabetes history of mother	Hypertension	Waist to height ratio	Urine nitrite

Abbreviation: LR, logistic regression; CART, classification and regression tree; GBM, gradient boosting machine; ANN, artificial neural network; RF, Random forest; SVM, Support vector machine.

We analyzed the importance of variables based on all models using the shapley additive explanations approach (Supplementary Table 4). As shown in Table 3. Among the top-10 variables across all methods were sweet flavor, urine glucose, age, heart rate, creatinine, waist circumference, uric acid, pulse pressure, insulin, and hypertension.

**Table 3:** Variable ranking based on the mean rank of all models based on shapley additive explanations approach.

Model		LR	CART	GBM	ANN	RF	SVM	Mean rank
Feature importance rank	Sweet flavor	3	2	1	4	1	3	2.33
	Urine glucose	5	1	3	6	2	1	3
	Age	2	4	2	5	4	2	3.17
	Heart rate	8	10	4	10	6	8	7.67
	Creatinine	7	13	6	9	9	6	8.33
	Waist circumference	4	20	11	7	11	4	9.5
	Uric acid	10	19	7	14	12	7	11.5
	Pulse pressure	16	7	10	11	10	20	12.33
	Insulin	12	8	14	15	18	13	13.33
	Hypertension	15	32	9	18	5	11	15

LR indicates logistic regression; CART, classification and regression tree; GBM, gradient boosting machine; ANN, artificial neural network; RF, Random forest; SVM, Support vector machine.

### Comparison of model performance

Table 4 presented the comparison results of machine learning algorithms. Using all available variables, all models for predicting risk of T2DM demonstrated strong predictive performance, with AUCs ranging between 0.811 and 0.872. The GBM model performed best (AUC = 0.872 with laboratory variables), and also presented better specificity (81.71%), positive predictive value (28.83%), and AUPR (0.546). In terms of accuracy and negative predictive value, the data showed that RF model remained strong predictive performance (85.90%, and 97.52% respectively). ANN model’s sensitivity was best among all models’. Using only non-laboratory data, such as BMI, age resulted in large declines in model performance. Moreover, the models with only non-laboratory data were also significantly better than the New Chinese Diabetes Risk Score^42^ based on statistical knowledge only using non-laboratory data (AUC = 0.728, p < 0.05) (Supplementary Figure 1).

**Table 4:** Performance of the machine-learning algorithms.

Lab	Model	AUC	Accuracy(%)	Sensitivity(%)	Specificity(%)	PPV(%)	NPV(%)	AUPR
**With lab**	LR	0.841 (0.825–0.858)	75.23	78.49	74.91	23.37	97.28	0.493
	CART	0.811 (0.793–0.829)	80.06	66.97	81.33	25.91	96.19	0.433
	GBM	0.872 (0.858–0.886)	81.20	76.04	81.71	28.83	97.22	0.546
	ANN	0.858 (0.842–0.873)	74.01	80.95	73.34	22.83	97.53	0.520
	RF	0.868 (0.854–0.883)	85.90	79.57	78.14	26.19	97.52	0.538
	SVM	0.835 (0.818–0.851)	76.42	74.65	76.59	23.71	96.88	0.490
**No lab**	LR	0.804 (0.787–0.821)	75.06	72.35	75.33	22.23	96.55	0.313
	CART	0.767 (0.749–0.784)	62.79	79.26	61.18	16.60	96.80	0.235
	GBM	0.817 (0.801–0.833)	70.28	78.96	69.43	20.11	97.13	0.345
	ANN	0.808 (0.791–0.825)	70.52	78.03	69.79	20.11	97.02	0.328
	RF	0.803 (0.786–0.820)	70.77	75.58	70.30	19.87	96.73	0.327
	SVM	0.800 (0.783–0.818)	76.46	70.51	77.04	23.03	96.40	0.316

Abbreviation: LR, logistic regression; CART, classification and regression tree; GBM, gradient boosting machine; ANN, artificial neural network; RF, Random forest; SVM, Support vector machine.

**Figure 2:**
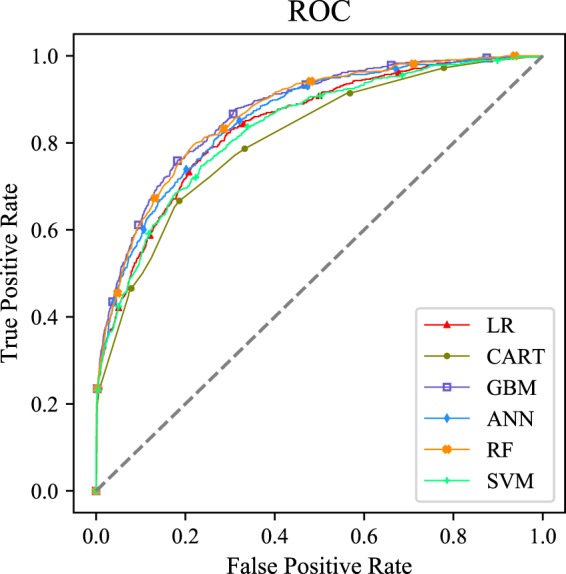
Receiver operating characteristic curve of different machine learning models. Abbreviation: LR, logistic regression; CART, classification and regression tree; GBM, gradient boosting machine; ANN, artificial neural network; RF, Random forest; SVM, Support vector machine.

**Figure 3:**
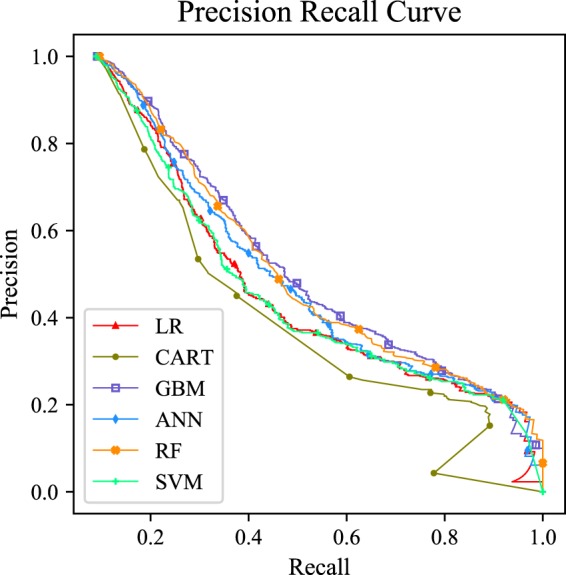
Precision recall curve of different machine learning models. Abbreviation: LR, logistic regression; CART, classification and regression tree; GBM, gradient boosting machine; ANN, artificial neural network; RF, Random forest; SVM, Support vector machine.

Figure 2 displayed the receiver operator characteristic curves of each model with all variables. This visualization revealed that the GBM performed similarly to RF model, and the two models exhibited greater superiority than the ANN model (*p* < *0.05*), with 0.872, 0.868 and 0.858 respectively. The above three models (GBM, RF and ANN) performed significantly better than CART (AUC = 0.11), LR (AUC = 0.841), and SVM (AUC = 0.835) (*p* < *0.05*). The area under precision recall curve also showed the same results (Figure 3).

### Model performance with a varying number of variables

**Figure 4:**
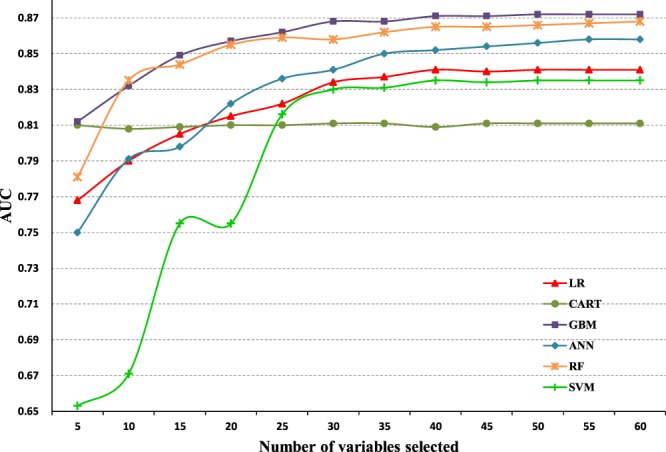
Performance variation of different models on a varying number of variables. LR indicates logistic regression; CART, classification and regression tree; GBM, gradient boosting machine; ANN, artificial neural network; RF, Random forest; SVM, Support vector machine.

In order to compare the performance of different models on a varying number of variables, the highest 5/10/15… ranked variables of each model were consecutively incorporated into each model. As shown in Figure 4. Overall, as the number of variables increased, the graph showed the increase of AUC values except the CART model. Before introducing with 30 variables, the LR, GBM, ANN, SVM, and RF models presented dramatic rise trends on the values of AUC. Performance of the five models plateaued when introduced 30 variables to each model. After that, all trends showed slight fluctuation, but the changes were modest. The CART model maintained a constant trend of AUC value.

## Discussion

Using machine learning methods, this study developed several risk assessment models for characterizing the risk of developing T2DM. High predictive performance was achieved by all models, with AUCs ranging from 0.811to 0.872. Compared to other models, the GBM model performed the best, with an AUC value of 0.872 (95% 0.858–0.886) and the models’ performance significantly better than the traditional risk score. In addition to common factors for diabetes, new important factors (urinary parameters) were not found in previous risk assessment methods, but determined by machine learning in our study. Our study demonstrated that machine learning technologies are uniquely positioned to identify significant risk factors in large-scale epidemiological studies.

To our knowledge, this is the first study to assess the importance of variables and characterize the risk of developing T2DM with use of different machine learning methods in a Chinese rural population. Our results were consistent with the previous findings. The New Chinese Diabetes Risk Score showed that sex, age, family history of diabetes, waist circumference, BMI, SBP were important risk factors^42^. Our results also revealed their prominent presence on the top-10 key factors for T2DM. Our data also indicated that obesity was a major risk factor for the development of T2DM^43^. The previous studies have demonstrated the significant role of boosting method in other medical fields, such as urinary tract infections^44^, hepatocellular carcinoma diagnosis^45^, prediction of hip fracture^46^. Our results confirmed the outstanding performance of the boosting method in the risk assessment of T2DM.

Identifying the key factors is of great clinical significance in the risk assessment of T2DM. The severity of T2DM is often estimated through a lot of factors in different aspects, including socio-demographic characteristics, anthropometric measures and laboratory test data. Given that the diversity and massive of factors in the development phase of T2DM, it is difficult to choose a specific number of variables for risk assessment. Compared to models with no laboratory data, the inclusion of laboratory data resulted in significant increase in the identification capabilities of models. This phenomenon shows that adding effective laboratory data can help identify the risk of T2DM patients. Our study also showed that the importance of different factors depended on the modeling technique. For LR, SVM and ANN models, the genetic factors and urinary indicators, such as diabetes history of mother/father, urine glucose, occupied center stage in the risk assessment of T2DM. Moreover, our results showed 30–35 variables were needed when the model performance reached a plateau, and the model performance would not be improved with too many variables. Over the past decade, the ability to collect data has become faster and cheaper, but we need to pay more attention to the model with too many features.

Data analysis techniques or model fitting ability are important in disease risk assessment and prediction. With the use of traditional statistical approaches, many risk scores and prediction models have been developed based on logistic regression. If the relationship among the data is linearly separable, traditional methods will fit better^47,48^. If not, such models may oversimplify complex relationships among factors with nonlinear interactions, leading to the potential loss of important relevant information. This suggests the important of choosing an appropriate model according to the characteristics of the data set. According to the data characteristics of the Henan Rural Cohort Study, our results showed the boosting model fit data best.

The performance of diagnosis model based on machine learning will be better if the number of training samples is large^49^. Compared to previous studies, the major strength of our study was the relatively large sample size including 36652 subjects from the rural population in China. Also, we compared the model performance from two aspects: the fixed number of variables and the dynamic number of variables, which confirmed models with several variables could perform no worse than the model with all variables^50^. Furthermore, the superiority and feasibility of nonparametric algorithms were proved compared with the model based on logistic regression.

However, several limitations should be worth mentioning. Firstly, the research findings were derived from a cross-sectional study without follow-up data; therefore, we may not be able to determine the causal and temporal associations. Secondly, we need to do future research with external validation and other machine learning methods to assess the model performance. In addition, it’s difficult to explain the inherent complexity of variable interactions and their impacts on outcomes due to the “black box” nature of machine learning methods.

In conclusion, using a series of machine learning models, we developed a data mining approach to characterize risk ofT2DM and compared the model performance from the fixed number of variables and the dynamic number of variables. Our results showed the advantage ability of machine learning to identify risk factors and predict outcomes across a wide range of data and an increasing number of variables, which leading to greater insights on disease risk factors with no prior assumption of causality.

### Data sharing statement

All relevant data are within the paper and its Supporting Information files. Contact to Dr. Chongjian Wang (tjwcj2005@126.com) for additional information regarding data access.

## Supplementary information

Supplementary information.
