# Supplementary material for: Machine learning for characterizing risk of type 2 diabetes mellitus in a rural Chinese population: the Henan Rural Cohort Study
Source: Sci Rep. 2020 Mar 10;10:4406. doi: 10.1038/s41598-020-61123-x (PMC7064542; doi:10.1038/s41598-020-61123-x)
Supplement: Supplementary file 1 — Supplementary information. [file 41598_2020_61123_MOESM1_ESM.docx]

Manuscript number: SREP-19-36134C

**Title：**

Machine learning for characterizing risk of type 2 diabetes mellitus in a rural Chinese population:

the Henan Rural Cohort Study

**Authors:**

Liying Zhang^a,b^, Yikang Wang^b^, Miaomiao Niu^b^, Chongjian Wang^b^, Zhenfei Wang^a*^

**Authors affiliations:**

^a^ School of Information Engineering, Zhengzhou University, Zhengzhou, Henan, PR China.

^b^ Department of Epidemiology and Biostatistics, College of Public Health, Zhengzhou University, Zhengzhou, Henan, PR China.

*** Correspondence author**

Dr. Zhenfei Wang

School of Information Engineering, Zhengzhou University

100 Kexue Avenue, Zhengzhou, 450001, Henan, PR China

Phone: +86 371 67783108;

Fax: +86 371 67783108

E-mail: iezfwang@zzu.edu.cn

**
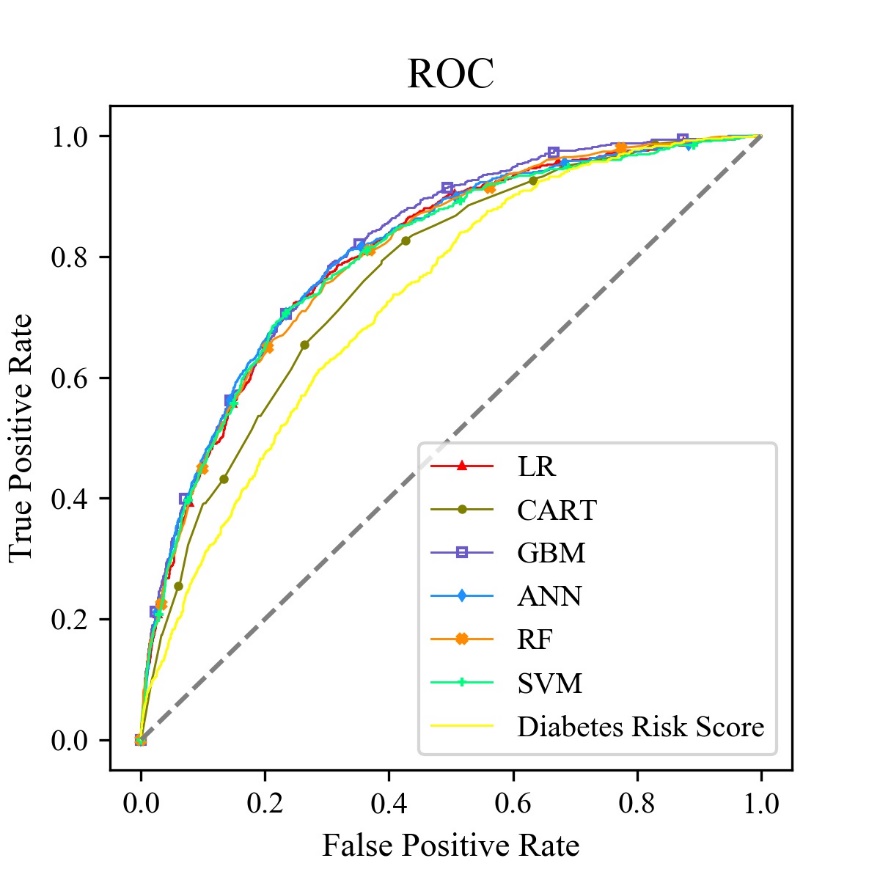
**

**Figure 1.** Receiver operating characteristic curve of different machine learning models and the New Chinese Diabetes Risk Score.

Abbreviation: LR, logistic regression; CART, classification and regression tree; GBM, gradient boosting machine; ANN, artificial neural network; RF, Random forest; SVM, Support vector machine.

Table 1. General characteristics of the study population

| **Variable** | **Total**  **( n=36652)** | **Non-T2DM**  **( n_1_=33296)** | **T2DM**  **( n_2_ =3356)** | ***P-Value*** |
| --- | --- | --- | --- | --- |
| Age (years) | 55.60±12.17 | 55.11±12.32 | 60.51±9.20 | <0.001 |
| Men, n (%) | 14375(39.22) | 13114(39.39) | 1261(37.54) | 0.040 |
| Education, n (%) |  |  |  | <0.001 |
| ≤ Primary school | 16432(44.83) | 14567(43.75) | 1865(55.57) |  |
| Middle school | 14614(39.87) | 13507(40.57) | 1107(32.99) |  |
| ≥ High school | 5606(15.30) | 5222(15.68) | 384(11.44) |  |
| Marry, n (%) |  |  |  | 0.027 |
| Married/cohabitating | 32927(89.84) | 29949(89.95) | 29877(88.74) |  |
| Divorced/widowed/unmarried | 3725(10.16) | 3347(10.05) | 378(11.26) |  |
| Average monthly individual income, n (%) |  |  |  | <0.001 |
| <1000 | 25111(68.51) | 22709(68.20) | 2402(71.57) |  |
| 1000~ | 8833(24.10) | 8083(24.28) | 750(22.35) |  |
| ≥2000 | 2708(7.39) | 2504(7.52) | 204(6.08) |  |
| Smoking, n (%) |  |  |  | <0.001 |
| Never | 26776 (73.05) | 24233(72.78) | 2543(75.77) |  |
| Light | 2037 (5.56) | 1885 (5.66) | 152 (4.53) |  |
| Moderate | 1632 (4.45) | 1510 (4.54) | 122 (3.64) |  |
| Heavy | 6207(16.93) | 5668 (17.02) | 539 (16.06) |  |
| Drinking, n (%) |  |  |  | 0.003 |
| Never | 28479 (77.70) | 25808 (77.51) | 2671 (79.59) |  |
| Light | 5063(13.81) | 4668 (14.02) | 395 (11.77) |  |
| Moderate | 1721 (4.70) | 1568 (4.71) | 153 (4.56) |  |
| Heavy | 1389 (3.79) | 1252 (3.76) | 137 (4.08) |  |
| High fat diet, ( ≥75g/day) | 7088(19.34) | 6544(19.65) | 544(16.21) | <0.001 |
| More vegetables and fruits, ( ≥500g/day) | 15389(42.99) | 14197(42.64) | 1192(35.52) | <0.001 |
| High Salt diet, ( ≥2g/day) | 6532(17.82) | 5919(17.78) | 613(18.27) | 0.481 |
| Salt flavor, n (%) |  |  |  | <0.001 |
| No | 10946(29.86) | 9838(29.55) | 1108(33.02) |  |
| Mild | 15758(42.99) | 14363(43.14) | 1395(41.57) |  |
| Middle | 9304(25.38) | 8491(25.50) | 813(24.23) |  |
| Heavy | 644(1.76) | 604(1.81) | 40(1.19) |  |
| Sweet flavor, n (%) |  |  |  | <0.001 |
| No | 15872(43.30) | 13495(40.53) | 2377(70.83) |  |
| Mild | 14217(38.79) | 13500(40.55) | 717(21.36) |  |
| Middle | 5720(15.61) | 5494(16.50) | 226(6.73) |  |
| Heavy | 843(2.30) | 807(2.42) | 36(1.07) |  |
| Sour flavor, n (%) |  |  |  |  |
| No | 15793(43.09) | 14380(43.19) | 1413(42.10) | 0.681 |
| Mild | 16097(43.92) | 14601(43.85) | 1496(44.58) |  |
| Middle | 4233(11.55) | 3836(11.52) | 397(11.83) |  |
| Heavy | 529(1.44) | 479(1.44) | 50(1.49) |  |
| Spicy flavor, n (%) |  |  |  | <0.001 |
| No | 15687(42.80) | 14079(42.28) | 1608(47.91) |  |
| Mild | 14385(39.25) | 13194(39.63) | 1191(35.49) |  |
| Middle | 5107(13.93) | 4670(14.03) | 437(13.02) |  |
| Heavy | 1473(4.02) | 1353(4.06) | 120(3.58) |  |
| Drinking tea frequently, n (%) | 5437(14.83) | 4944(14.85) | 493(14.69) | 0.805 |
| Physical activity, n (%) |  |  |  | <0.001 |
| Low | 11761(32.09) | 10465(31.43) | 1296(38.62) |  |
| Moderate | 13988(38.16) | 12776(38.37) | 1212(36.11) |  |
| High | 10903(29.75) | 10055(30.20) | 848(25.27) |  |
| Waist circumference (cm) | 84.13±10.33 | 83.62±10.22 | 89.32±10.01 | <0.001 |
| Hip circumference (cm) | 94.51±6.85 | 94.39±16.81 | 95.75±17.12 | <0.001 |
| Body mass index (kg/m**^2^**) | 24.85±3.53 | 24.72±3.49 | 26.20±3.62 | <0.001 |
| Height(cm) | 159.68±8.18 | 159.75±8.17 | 158.99±8.30 | <0.001 |
| Weight(kg) | 63.52±11.06 | 63.23±10.97 | 66.40±11.56 | <0.001 |
| Waist to hip ratio | 0.89±0.07 | 0.88±0.07 | 0.93±0.07 | <0.001 |
| Waist to height ratio | 0.53±0.07 | 0.52±0.06 | 0.56±0.06 | <0.001 |
| Systolic blood pressure (mm Hg) | 125.89±19.96 | 125.09±19.81 | 133.80±19.73 | <0.001 |
| Diastolic blood pressure (mm Hg) | 77.64±11.61 | 77.37±11.63 | 80.35±11.10 | <0.001 |
| Pulse pressure (mm Hg) | 48.25±13.08 | 47.72±12.85 | 53.45±14.22 | <0.001 |
| Heart rate (beats/min) | 75.72±11.12 | 75.34±10.94 | 79.54±12.13 | <0.001 |
| Basal energy expenditure (kCal) | 1338.68±191.11 | 1338.11±190.59 | 1344.37±196.20 | 0.077 |
| Body fat percent (%) | 30.12±6.65 | 29.87±6.64 | 32.52±6.26 | <0.001 |
| Basal [metabolic](javascript:;) [rate](javascript:;) (kCal) | 1374.42±212.57 | 1371.40±211.28 | 1404.31±222.86 | <0.001 |
| Visceral fat index | 9.48±4.57 | 9.28±4.49 | 11.50±4.92 | <0.001 |
| Total cholesterol (mmol/l) | 4.75±0.97 | 4.72±0.95 | 5.01±1.11 | <0.001 |
| Triglyceride (mmol/l) | 1.68±1.12 | 1.64±1.07 | 2.13±1.44 | <0.001 |
| HDL-C (mmol/l) | 1.32±0.33 | 1.33±0.33 | 1.23±0.32 | <0.001 |
| LDL-C (mmol/l) | 2.87±0.81 | 2.85±0.80 | 3.06±0.93 | <0.001 |
| Insulin (ug/l) | 10.85±5.30 | 10.69±5.04 | 12.51±7.19 | <0.001 |
| Creatinine (umol/L) | 62.07±14.00 | 62.31±13.75 | 59.61±16.08 | <0.001 |
| Urea (mmol/l) | 5.35±1.51 | 5.34±1.51 | 5.50±1.58 | <0.001 |
| Uric acid (umol/L) | 286.50±79.29 | 287.77±79.19 | 273.87±79.22 | <0.001 |
| Urine white blood cells, n (%) | 4115(11.23) | 3700(11.11) | 415(12.37) | 0.028 |
| Urine ketone bodies, n (%) | 321(0.88) | 245(0.74) | 76(2.26) | <0.001 |
| Urine nitrite, n (%) | 897(2.45) | 753(2.26) | 144(4.29) | <0.001 |
| Urine urobilinogen, n (%) | 3597(9.81) | 3308(9.94) | 289(8.61) | 0.014 |
| Urine bilirubin, n (%) | 681(1.86) | 601(1.81) | 80(2.38) | 0.018 |
| Urinary protein, n (%) | 1087(2.97) | 797(2.39) | 290(8.64) | <0.001 |
| Urine glucose, n (%) | 915(2.50) | 125(0.38) | 790(23.54) | <0.001 |
| Urine specific gravity, n (%) | 31716(86.53) | 28748(86.34) | 2968(88.44) | <0.001 |
| Urine latent blood, n (%) | 5036(13.74) | 4776(14.34) | 260(7.75) | <0.001 |
| Urine PH, n (%) | 24671(67.31) | 22261(66.86) | 2410(71.81) | <0.001 |
| Urine vitamin C, n (%) | 20069(54.76) | 18365(55.16) | 1704(50.77) | <0.001 |
| Hypertension, n (%) | 11943(32.58) | 10225(30.71) | 1718(51.19) | <0.001 |
| Coronary heart disease, n (%) | 1620(4.42) | 1368(4.11) | 252(7.51) | <0.001 |
| T2DM history of mother, n (%) | 1070(2.92) | 813(2.44) | 257(7.66) | <0.001 |
| T2DM history of father, n (%) | 532(1.45) | 432(1.30) | 100(1.45) | <0.001 |
| Hypertension history of mother, n (%) | 5036(13.74) | 45852(13.77) | 451(13.44) | 0.595 |
| Hypertension history of father, n (%) | 3473(9.48) | 3136(9.42) | 337(10.04) | 0.240 |
| Dyslipidemia history of mother, n (%) | 863(2.35) | 806(2.42) | 57(1.70) | 0.009 |
| Dyslipidemia history of father, n (%) | 603(1.65) | 547(1.64) | 56(1.67) | 0.911 |
| CHD history of mother, n (%) | 1953(5.33) | 1812(5.44) | 141(4.20) | 0.002 |
| CHD history of father, n (%) | 1251(3.41) | 1162(3.49) | 89(2.65) | 0.011 |

Abbreviations: SD, standard deviation; HDL-C, high-density lipoprotein cholesterol; LDL-C, low-density lipoprotein cholesterol; T2DM, type 2 diabetes mellitus; CHD, coronary heart disease.

Table 2: Using Grid Search Parameter Optimization

| Algorithm | Parameters tuned | Grid Serach Script | AUC range | Optimal performance |
| --- | --- | --- | --- | --- |
| Classification and regression tree (CART) | max_depth (1, 3, 5, 7,10)  min_samples_leaf=np.arange(1, 11)  max_features (1, 3, 5, 10) | scoring = {'AUC': 'roc_auc'}  max_depth=[1,3,5,7,10]  min_samples_leaf=np.arange(1, 11)  max_leaf_nodes =np.arange(1, 11)  param_grid = {'max_depth': max_depth,  'min_samples_leaf': min_samples_leaf, 'max_leaf_nodes': max_leaf_nodes  clf = GridSearchCV(DecisionTreeClassifier (), param_grid, scoring=scoring, cv=10, refit='AUC', return_train_score=True) | 0.610-0.811 | 0.811  max_depth=7  min_samples_leaf=7  min_samples_leaf=2 |
| Random Forest(RF) | n_estimators (100, 300, 500,1000)  max_depth (1, 3, 5,10)  max_features (1, 3, 5, 10) | scoring = {'AUC': 'roc_auc'}  param_grid = [ {'n_estimators': [100,300,500,1000],'max_depth':[1,3,5,10], 'max_features':[1,3,5,10] } ]  clf = GridSearchCV(RandomForestClassifier (), param_grid, scoring=scoring, cv=10, refit='AUC', return_train_score=True) | 0.819-0.869 | 0.868  n_estimators=500  max_features=5 |
| Gradient boosting machine (GBM) | n_estimators (100, 300, 500,1000)  max_depth (1, 3, 5,10)  max_features (1, 3, 5, 10) | scoring = {'AUC': 'roc_auc'}  param_grid = [ {'n_estimators': [100,300,500,1000],'max_depth':[1,3,5,10], 'max_features':[1,3,5,10] } ]  clf = GridSearchCV(GradientBoostingClassifier(), param_grid, scoring=scoring, cv=10, refit='AUC', return_train_score=True) | 0.853-0.875 | 0.872  n_estimators=300  max_features=5 |
| Artificial neural networks (ANN) | hidden_layer_sizes=np.arange(1, 11)  max_iter(1000, 2000, 3000)  learning_rate_init (0.0001, 0.001, 0.01,0.1)  learning_rate{‘constant’, ‘invscaling’, ‘adaptive’} | scoring = {'AUC': 'roc_auc'}  hidden_layer_sizes=np.arange(1, 11)  max_iter=[1000, 2000, 3000]  learning_rate_init=[0.0001, 0.001, 0.01,0.1]  learning_rate=[‘constant’, ‘invscaling’, ‘adaptive’]  param_grid = {' hidden_layer_sizes ': hidden_layer_sizes,  ' max_iter ': max_iter , ' learning_rate_init ': learning_rate_init, ' learning_rate ': learning_rate  clf = GridSearchCV(MLPClassifier (), param_grid, scoring=scoring, cv=10, refit='AUC', return_train_score=True) | 0.5-0.860 | 0.858  hidden_layer_sizes=5  max_iter=2000  learning_rate_init=0.0001  learning_rate= 'invscaling' |
| Support vector machine (SVM) | kernel='linear'  C (0.001, 0.01, 0.1, 1, 2, 3, 5, 7, 10)  The implementation of SVM is based on libsvm. The fit time scales at least quadratically with the number of samples  and may be impractical beyond tens of thousands of samples. Therefore, for large datasets consider using kernel='linear'. | scoring = {'AUC': 'roc_auc'}  param_grid = [scoring = {'AUC': 'roc_auc'}  param_grid = [ { 'C':[ 0.001, 0.01, 0.1, 1, 2, 3, 5, 7, 10] } ]  clf = GridSearchCV(SVC (), param_grid, scoring=scoring, cv=10, refit='AUC', return_train_score=True) | 0.846-0.855 | 0.854  C=0.01 |
| Logistic Regression(LR) |  |  |  | Default settings |

Table 3. Ranking of important variables for machine-learning methods

| **Variables** | **VI** | **GBM Rank** | **VI** | **RF**  **Rank** | **Coefficient** | **LR Rank** | **VI** | **CART Rank** | **Weight** | **ANN**  **Rank** | **Coefficient** | **SVM**  **Rank** |
| --- | --- | --- | --- | --- | --- | --- | --- | --- | --- | --- | --- | --- |
| age | 0.032773 | 6 | 0.027673 | 4 | 0.053133 | 18 | 0.022104 | 5 | 0.060058 | 21 | 0.006283 | 23 |
| Gender | 0.000674 | 55 | 0.002456 | 54 | -0.319278 | 57 | 0.000000 | 50 | 0.103588 | 8 | -0.016490 | 50 |
| Education | 0.015805 | 19 | 0.014732 | 33 | 0.016712 | 30 | 0.000000 | 49 | 0.042722 | 46 | 0.001713 | 30 |
| Marry | 0.001773 | 46 | 0.004117 | 47 | -0.225442 | 56 | 0.000000 | 46 | 0.074657 | 13 | -0.021922 | 53 |
| Average monthly individual income | 0.008287 | 27 | 0.008715 | 38 | 0.022656 | 28 | 0.000000 | 47 | 0.049475 | 36 | -0.000346 | 35 |
| Smoking | 0.000926 | 52 | 0.004495 | 46 | -0.075427 | 43 | 0.000000 | 51 | 0.046848 | 38 | -0.012668 | 47 |
| drinking | 0.000792 | 54 | 0.004764 | 44 | -0.083841 | 47 | 0.000000 | 59 | 0.050099 | 32 | -0.006358 | 43 |
| High fat diet | 0.004927 | 33 | 0.005896 | 41 | 0.080760 | 16 | 0.000000 | 53 | 0.040956 | 50 | 0.011091 | 16 |
| Vegetables | 0.034127 | 5 | 0.020686 | 15 | -0.218722 | 55 | 0.000000 | 38 | 0.051173 | 29 | -0.025181 | 56 |
| Salt | 0.005783 | 29 | 0.005622 | 42 | 0.039388 | 21 | 0.000000 | 52 | 0.054246 | 27 | 0.008288 | 20 |
| Salt flavor | 0.014339 | 21 | 0.013513 | 36 | -0.085499 | 48 | 0.001966 | 18 | 0.064574 | 17 | -0.013107 | 49 |
| Sweet flavor | 0.113104 | 2 | 0.062444 | 2 | -0.801974 | 59 | 0.261521 | 2 | 0.186086 | 5 | -0.087583 | 59 |
| Sour flavor | 0.025797 | 9 | 0.017499 | 30 | 0.117040 | 13 | 0.060777 | 3 | 0.072702 | 14 | 0.013340 | 15 |
| Spicy flavor | 0.015776 | 20 | 0.013697 | 35 | 0.032127 | 26 | 0.000000 | 41 | 0.061638 | 20 | 0.003540 | 29 |
| Tea | 0.001572 | 48 | 0.004050 | 48 | 0.038334 | 22 | 0.000011 | 34 | 0.044273 | 42 | 0.000816 | 32 |
| Physical activity | 0.019245 | 14 | 0.016990 | 31 | -0.075689 | 45 | 0.000925 | 22 | 0.040024 | 53 | -0.012893 | 48 |
| Height | 0.001701 | 47 | 0.018119 | 29 | -0.015427 | 38 | 0.000771 | 23 | 0.058513 | 23 | 0.004707 | 25 |
| Waist circumference | 0.012195 | 22 | 0.023895 | 11 | 0.052750 | 19 | 0.001804 | 20 | 0.028327 | 59 | 0.006086 | 24 |
| Hip circumference | 0.001520 | 49 | 0.018531 | 26 | -0.059564 | 41 | 0.000400 | 25 | 0.043480 | 45 | -0.008096 | 44 |
| Weight | 0.001990 | 44 | 0.018521 | 27 | 0.062463 | 17 | 0.000017 | 32 | 0.056960 | 24 | -0.000835 | 38 |
| Basal energy expenditure | 0.002703 | 38 | 0.019709 | 19 | 0.000689 | 32 | 0.002403 | 15 | 0.072604 | 15 | 0.000103 | 33 |
| Body fat percent | 0.003771 | 35 | 0.019859 | 17 | -0.014232 | 36 | 0.003563 | 13 | 0.054424 | 26 | -0.003362 | 40 |
| B[asal](javascript:;) [metabolic](javascript:;) [rate](javascript:;) | 0.002246 | 41 | 0.019093 | 23 | -0.001534 | 34 | 0.000082 | 30 | 0.062613 | 18 | -0.000135 | 34 |
| Visceral fat index | 0.009277 | 26 | 0.019330 | 22 | 0.022699 | 27 | 0.000100 | 29 | 0.040800 | 51 | 0.004106 | 28 |
| Body mass index | 0.002826 | 37 | 0.019835 | 18 | -0.075607 | 44 | 0.000144 | 28 | 0.043592 | 44 | 0.010046 | 17 |
| Waist to hip ratio | 0.039014 | 3 | 0.033532 | 3 | 0.017373 | 29 | 0.042495 | 4 | 0.050785 | 31 | 0.015226 | 14 |
| Waist to height ratio | 0.019448 | 12 | 0.024061 | 10 | -0.015035 | 37 | 0.011043 | 7 | 0.040635 | 52 | -0.005647 | 42 |
| Systolic blood pressure | 0.006815 | 28 | 0.021423 | 14 | -0.001346 | 33 | 0.000004 | 35 | 0.091009 | 9 | -0.000454 | 36 |
| Diastolic blood pressure | 0.005482 | 30 | 0.019049 | 24 | -0.015957 | 39 | 0.001205 | 21 | 0.046094 | 39 | -0.002127 | 39 |
| Heart rate | 0.019414 | 13 | 0.026568 | 7 | 0.032675 | 25 | 0.007974 | 10 | 0.035748 | 56 | 0.004308 | 26 |
| Pulse pressure | 0.012182 | 23 | 0.023115 | 12 | 0.014611 | 31 | 0.008589 | 9 | 0.058900 | 22 | 0.001673 | 31 |
| Total cholesterol | 0.001938 | 45 | 0.019423 | 20 | -0.120470 | 49 | 0.000013 | 33 | 0.049810 | 34 | -0.011247 | 46 |
| Triglyceride | 0.017385 | 16 | 0.026026 | 9 | 0.178420 | 11 | 0.002054 | 17 | 0.049432 | 37 | 0.024876 | 11 |
| High-density lipoprotein cholesterol | 0.005084 | 32 | 0.020473 | 16 | -0.562110 | 58 | 0.000022 | 31 | 0.087471 | 11 | -0.046609 | 58 |
| Low-density lipoprotein cholesterol | 0.002045 | 43 | 0.019407 | 21 | 0.283393 | 8 | 0.000606 | 24 | 0.062198 | 19 | 0.031577 | 8 |
| Uric acid | 0.010173 | 25 | 0.027137 | 6 | -0.003686 | 35 | 0.002781 | 14 | 0.045832 | 40 | -0.000670 | 37 |
| Creatinine | 0.018852 | 15 | 0.027259 | 5 | -0.030727 | 40 | 0.004954 | 12 | 0.039519 | 54 | -0.003860 | 41 |
| Urea | 0.002222 | 42 | 0.018284 | 28 | 0.087078 | 14 | 0.000003 | 36 | 0.044350 | 41 | 0.009874 | 18 |
| Insulin | 0.016322 | 18 | 0.026220 | 8 | 0.048836 | 20 | 0.011010 | 8 | 0.051138 | 30 | 0.006402 | 22 |
| Urine white blood cells | 0.004888 | 34 | 0.005541 | 43 | 0.034700 | 23 | 0.007251 | 11 | 0.029429 | 58 | 0.007031 | 21 |
| Urine ketone bodies | 0.000832 | 53 | 0.001661 | 56 | 0.383984 | 5 | 0.000000 | 60 | 0.146849 | 7 | 0.106000 | 5 |
| Urine nitrite | 0.002422 | 40 | 0.002901 | 52 | 0.217964 | 10 | 0.000000 | 44 | 0.041308 | 49 | 0.030811 | 10 |
| Urine urobilinogen | 0.001146 | 51 | 0.003184 | 51 | -0.200670 | 54 | 0.000000 | 43 | 0.050090 | 33 | -0.030079 | 57 |
| Urine bilirubin | 0.000055 | 60 | 0.001203 | 58 | -0.062915 | 42 | 0.000000 | 57 | 0.041719 | 47 | 0.004139 | 27 |
| Urinary protein | 0.025325 | 11 | 0.013880 | 34 | 1.219000 | 3 | 0.002274 | 16 | 0.252705 | 3 | 0.171002 | 2 |
| Urine glucose | 0.296615 | 1 | 0.131963 | 1 | 4.844508 | 1 | 0.526690 | 1 | 0.928923 | 1 | 2.591139 | 1 |
| Urine specific gravity | 0.003176 | 36 | 0.004009 | 49 | 0.132522 | 12 | 0.000000 | 42 | 0.056177 | 25 | 0.017762 | 12 |
| Urine latent blood | 0.016383 | 17 | 0.006719 | 40 | -0.849164 | 60 | 0.000000 | 40 | 0.210358 | 4 | -0.090452 | 60 |
| Urine PH | 0.025966 | 8 | 0.016445 | 32 | 0.273014 | 9 | 0.000348 | 26 | 0.070768 | 16 | 0.031220 | 9 |
| Urine vitamin C | 0.031980 | 7 | 0.018736 | 25 | -0.165845 | 52 | 0.000000 | 39 | 0.049600 | 35 | -0.022417 | 54 |
| Hypertension | 0.034583 | 4 | 0.022657 | 13 | 0.372392 | 6 | 0.000000 | 37 | 0.090989 | 10 | 0.059859 | 6 |
| CHD | 0.010319 | 24 | 0.007084 | 39 | 0.349061 | 7 | 0.000243 | 27 | 0.078948 | 12 | 0.049545 | 7 |
| Hypertension history of father | 0.001442 | 50 | 0.003284 | 50 | 0.084701 | 15 | 0.000000 | 55 | 0.041595 | 48 | 0.009665 | 19 |
| Hypertension history of mother | 0.005470 | 31 | 0.004545 | 45 | -0.080901 | 46 | 0.000000 | 45 | 0.031295 | 57 | -0.009656 | 45 |
| Dyslipidemia history of father | 0.000064 | 59 | 0.000953 | 60 | 0.033351 | 24 | 0.000000 | 54 | 0.022224 | 60 | 0.017539 | 13 |
| Dyslipidemia history of mother | 0.000330 | 57 | 0.001304 | 57 | -0.143908 | 50 | 0.000000 | 58 | 0.053470 | 28 | -0.023006 | 55 |
| T2DM history of father | 0.002590 | 39 | 0.002477 | 53 | 0.679958 | 4 | 0.001899 | 19 | 0.173163 | 6 | 0.115707 | 4 |
| T2DM history of mother | 0.025373 | 10 | 0.012375 | 37 | 1.370611 | 2 | 0.011952 | 6 | 0.290040 | 2 | 0.165632 | 3 |
| CHD history of father | 0.000113 | 58 | 0.001150 | 59 | -0.156613 | 51 | 0.000000 | 56 | 0.044040 | 43 | -0.019293 | 51 |
| CHD history of mother | 0.000623 | 56 | 0.001709 | 55 | -0.188563 | 53 | 0.000000 | 48 | 0.038959 | 55 | -0.019952 | 52 |

Abbreviations: T2DM, type 2 diabetes mellitus; CHD, coronary heart disease; VI, Variable importance; LR, logistic regression; CART, classification and regression tree; RF, Random forest; ANN, artificial neural network; SVM, support vector machine.

Table 4. Ranking of important variables for machine-learning methods based on shapley additive explanations approach.

| **Variables** | **Shap_values** | **GBM Rank** | **Shap_values** | **RF**  **Rank** | **Shap_values** | **LR Rank** | **Shap_values** | **CART Rank** | **Shap_values** | **ANN**  **Rank** | **Shap_values** | **SVM**  **Rank** |
| --- | --- | --- | --- | --- | --- | --- | --- | --- | --- | --- | --- | --- |
| age | 0.435096 | 2 | 0.018778 | 4 | 0.524620 | 2 | 0.024033 | 4 | 0.042552 | 5 | 0.059111 | 2 |
| Gender | 0.022907 | 40 | 0.000861 | 53 | 0.140143 | 19 | 0.000000 | 50 | 0.003780 | 34 | 0.007371 | 34 |
| Education | 0.034609 | 30 | 0.005928 | 20 | 0.010898 | 51 | 0.000000 | 49 | 0.000026 | 58 | 0.000773 | 55 |
| Marry | 0.014357 | 50 | 0.001230 | 44 | 0.039807 | 36 | 0.000000 | 46 | 0.002244 | 36 | 0.003796 | 40 |
| Average monthly individual income | 0.045961 | 28 | 0.002338 | 39 | 0.011748 | 48 | 0.000000 | 47 | 0.000102 | 56 | 0.000311 | 58 |
| Smoking | 0.011533 | 52 | 0.001067 | 47 | 0.063476 | 33 | 0.000000 | 51 | 0.003781 | 33 | 0.010464 | 30 |
| drinking | 0.008135 | 53 | 0.000973 | 49 | 0.036274 | 37 | 0.000000 | 59 | 0.002178 | 37 | 0.004073 | 39 |
| High fat diet | 0.028735 | 36 | 0.001539 | 42 | 0.024411 | 41 | 0.000000 | 53 | 0.001150 | 44 | 0.003793 | 41 |
| Vegetables | 0.107523 | 18 | 0.009040 | 13 | 0.102953 | 25 | 0.000000 | 33 | 0.009398 | 22 | 0.010599 | 29 |
| Salt | 0.030944 | 34 | 0.001453 | 43 | 0.011634 | 49 | 0.000000 | 52 | 0.000655 | 45 | 0.001180 | 52 |
| Salt flavor | 0.061660 | 24 | 0.004013 | 27 | 0.050131 | 34 | 0.000355 | 24 | 0.004126 | 32 | 0.008196 | 31 |
| Sweet flavor | 0.548487 | 1 | 0.044220 | 1 | 0.522688 | 3 | 0.075961 | 2 | 0.045763 | 4 | 0.056449 | 3 |
| Sour flavor | 0.107680 | 17 | 0.005494 | 24 | 0.073745 | 30 | 0.010639 | 6 | 0.004994 | 29 | 0.008093 | 32 |
| Spicy flavor | 0.057026 | 25 | 0.003492 | 32 | 0.015658 | 46 | 0.000000 | 40 | 0.001325 | 42 | 0.001890 | 46 |
| Tea | 0.020691 | 41 | 0.001120 | 46 | 0.010665 | 52 | 0.000110 | 29 | 0.000642 | 46 | 0.000170 | 59 |
| Physical activity | 0.077892 | 20 | 0.005687 | 22 | 0.046179 | 35 | 0.000921 | 17 | 0.004577 | 31 | 0.007534 | 33 |
| Height | 0.031368 | 32 | 0.002290 | 40 | 0.113766 | 22 | 0.000802 | 18 | 0.004642 | 30 | 0.031528 | 9 |
| Waist circumference | 0.118205 | 11 | 0.009978 | 11 | 0.433219 | 4 | 0.000609 | 20 | 0.033192 | 7 | 0.052209 | 4 |
| Hip circumference | 0.011805 | 51 | 0.003052 | 35 | 0.318346 | 6 | 0.000000 | 38 | 0.021744 | 13 | 0.044090 | 5 |
| Weight | 0.018310 | 43 | 0.002915 | 36 | 0.547476 | 1 | 0.000000 | 41 | 0.061281 | 1 | 0.002942 | 43 |
| Basal energy expenditure | 0.025396 | 37 | 0.003685 | 30 | 0.112933 | 23 | 0.001009 | 15 | 0.002164 | 38 | 0.014617 | 21 |
| Body fat percent | 0.014858 | 48 | 0.004388 | 25 | 0.072563 | 32 | 0.001003 | 16 | 0.015253 | 17 | 0.019581 | 17 |
| B[asal](javascript:;) [metabolic](javascript:;) [rate](javascript:;) | 0.015792 | 46 | 0.002712 | 37 | 0.247411 | 9 | 0.000130 | 28 | 0.032699 | 8 | 0.031126 | 10 |
| Visceral fat index | 0.061748 | 23 | 0.007121 | 14 | 0.073086 | 31 | 0.000211 | 27 | 0.017347 | 16 | 0.018876 | 19 |
| Body mass index | 0.019935 | 42 | 0.004214 | 26 | 0.215630 | 11 | 0.000078 | 30 | 0.023458 | 12 | 0.024466 | 12 |
| Waist to hip ratio | 0.223351 | 5 | 0.019312 | 3 | 0.000767 | 59 | 0.029717 | 3 | 0.000086 | 57 | 0.000889 | 53 |
| Waist to height ratio | 0.051559 | 26 | 0.011324 | 8 | 0.000638 | 60 | 0.014094 | 5 | 0.000022 | 59 | 0.000381 | 56 |
| Systolic blood pressure | 0.029223 | 35 | 0.005593 | 23 | 0.021736 | 42 | 0.000000 | 39 | 0.057604 | 2 | 0.006763 | 35 |
| Diastolic blood pressure | 0.067171 | 22 | 0.003139 | 34 | 0.146336 | 17 | 0.000364 | 23 | 0.048739 | 3 | 0.019583 | 16 |
| Heart rate | 0.227864 | 4 | 0.012603 | 6 | 0.283780 | 8 | 0.005649 | 10 | 0.026404 | 10 | 0.036218 | 8 |
| Pulse pressure | 0.118768 | 10 | 0.010083 | 10 | 0.149381 | 16 | 0.008828 | 7 | 0.024071 | 11 | 0.017513 | 20 |
| Total cholesterol | 0.024422 | 39 | 0.003533 | 31 | 0.087082 | 26 | 0.000000 | 37 | 0.001426 | 41 | 0.011720 | 26 |
| Triglyceride | 0.117561 | 12 | 0.011606 | 7 | 0.137670 | 20 | 0.001341 | 14 | 0.011453 | 20 | 0.019556 | 18 |
| High-density lipoprotein cholesterol | 0.109878 | 15 | 0.005698 | 21 | 0.144736 | 18 | 0.000000 | 36 | 0.008557 | 24 | 0.012321 | 22 |
| Low-density lipoprotein cholesterol | 0.035251 | 29 | 0.003695 | 29 | 0.172359 | 14 | 0.000341 | 25 | 0.005835 | 27 | 0.021065 | 14 |
| Uric acid | 0.171754 | 7 | 0.009116 | 12 | 0.231052 | 10 | 0.000744 | 19 | 0.019885 | 14 | 0.038557 | 7 |
| Creatinine | 0.186040 | 6 | 0.010737 | 9 | 0.313035 | 7 | 0.001725 | 13 | 0.028352 | 9 | 0.039618 | 6 |
| Urea | 0.049482 | 27 | 0.002632 | 38 | 0.107728 | 24 | 0.000063 | 31 | 0.008366 | 25 | 0.011825 | 24 |
| Insulin | 0.115156 | 14 | 0.006644 | 18 | 0.192080 | 12 | 0.006149 | 8 | 0.018789 | 15 | 0.024308 | 13 |
| Urine white blood cells | 0.016482 | 45 | 0.001897 | 41 | 0.008977 | 54 | 0.003139 | 11 | 0.000308 | 52 | 0.000835 | 54 |
| Urine ketone bodies | 0.002084 | 56 | 0.000588 | 55 | 0.006882 | 55 | 0.000000 | 60 | 0.000255 | 54 | 0.002030 | 45 |
| Urine nitrite | 0.014439 | 49 | 0.001145 | 45 | 0.011335 | 50 | 0.000000 | 44 | 0.000528 | 50 | 0.001669 | 47 |
| Urine urobilinogen | 0.007466 | 55 | 0.000929 | 51 | 0.034553 | 38 | 0.000000 | 43 | 0.001532 | 40 | 0.005104 | 36 |
| Urine bilirubin | 0.000000 | 60 | 0.000403 | 56 | 0.001480 | 57 | 0.000000 | 57 | 0.000140 | 55 | 0.000079 | 60 |
| Urinary protein | 0.074823 | 21 | 0.006140 | 19 | 0.079210 | 29 | 0.002094 | 12 | 0.006218 | 26 | 0.011315 | 27 |
| Urine glucose | 0.393562 | 3 | 0.044122 | 2 | 0.370977 | 5 | 0.077632 | 1 | 0.034869 | 6 | 0.209847 | 1 |
| Urine specific gravity | 0.024637 | 38 | 0.000858 | 54 | 0.030847 | 40 | 0.000000 | 42 | 0.001675 | 39 | 0.004650 | 37 |
| Urine latent blood | 0.145283 | 8 | 0.003801 | 28 | 0.191172 | 13 | 0.000000 | 35 | 0.014720 | 19 | 0.019996 | 15 |
| Urine PH | 0.115693 | 13 | 0.006720 | 16 | 0.115405 | 21 | 0.000327 | 26 | 0.010182 | 21 | 0.011968 | 23 |
| Urine vitamin C | 0.108421 | 16 | 0.006824 | 15 | 0.079799 | 28 | 0.000000 | 34 | 0.005480 | 28 | 0.011814 | 25 |
| Hypertension | 0.143999 | 9 | 0.015962 | 5 | 0.165279 | 15 | 0.000000 | 32 | 0.015184 | 18 | 0.024895 | 11 |
| CHD | 0.031106 | 33 | 0.003315 | 33 | 0.031549 | 39 | 0.000495 | 21 | 0.002881 | 35 | 0.004301 | 38 |
| Hypertension history of father | 0.007975 | 54 | 0.000931 | 50 | 0.014969 | 47 | 0.000000 | 55 | 0.000642 | 47 | 0.001572 | 49 |
| Hypertension history of mother | 0.032219 | 31 | 0.000913 | 52 | 0.018305 | 44 | 0.000000 | 45 | 0.000620 | 48 | 0.001632 | 48 |
| Dyslipidemia history of father | 0.001192 | 58 | 0.000318 | 60 | 0.001102 | 58 | 0.000000 | 54 | 0.000012 | 60 | 0.000351 | 57 |
| Dyslipidemia history of mother | 0.001489 | 57 | 0.000345 | 58 | 0.005478 | 56 | 0.000000 | 58 | 0.000297 | 53 | 0.001257 | 51 |
| T2DM history of father | 0.017819 | 44 | 0.001024 | 48 | 0.019160 | 43 | 0.000386 | 22 | 0.001208 | 43 | 0.003529 | 42 |
| T2DM history of mother | 0.088874 | 19 | 0.006658 | 17 | 0.085889 | 27 | 0.005892 | 9 | 0.009083 | 23 | 0.011036 | 28 |
| CHD history of father | 0.000499 | 59 | 0.000332 | 59 | 0.009303 | 53 | 0.000000 | 56 | 0.000499 | 51 | 0.001482 | 50 |
| CHD history of mother | 0.015047 | 47 | 0.000368 | 57 | 0.017538 | 45 | 0.000000 | 48 | 0.000568 | 49 | 0.002085 | 44 |

Abbreviations: T2DM, type 2 diabetes mellitus; CHD, coronary heart disease; VI, Variable importance; LR, logistic regression; CART, classification and regression tree; RF, Random forest; ANN, artificial neural network; SVM, support vector machine.
